# Single Unpurified Breast Tumor-Initiating Cells from Multiple Mouse Models Efficiently Elicit Tumors in Immune-Competent Hosts

**DOI:** 10.1371/journal.pone.0058151

**Published:** 2013-03-26

**Authors:** Natasza A. Kurpios, Adele Girgis-Gabardo, Robin M. Hallett, Stephen Rogers, David W. Gludish, Lisa Kockeritz, James Woodgett, Robert Cardiff, John A. Hassell

**Affiliations:** 1 Centre for Functional Genomics, Department of Biochemistry and Biomedical Sciences, McMaster University, Hamilton, Ontario, Canada; 2 Department of Medical Biophysics, University of Toronto, Toronto, Ontario, Canada; 3 Department of Pathology, University of California Davis, Davis, California, United States of America; Huntsman Cancer Institute, University of Utah, United States of America

## Abstract

The tumor-initiating cell (TIC) frequency of bulk tumor cell populations is one of the criteria used to distinguish malignancies that follow the cancer stem cell model from those that do not. However, tumor-initiating cell frequencies may be influenced by experimental conditions and the extent to which tumors have progressed, parameters that are not always addressed in studies of these cells. We employed limiting dilution cell transplantation of minimally manipulated tumor cells from mammary tumors of several transgenic mouse models to determine their tumor-initiating cell frequency. We determined whether the tumors that formed following tumor cell transplantation phenocopied the primary tumors from which they were isolated and whether they could be serially transplanted. Finally we investigated whether propagating primary tumor cells in different tissue culture conditions affected their resident tumor-initiating cell frequency. We found that tumor-initiating cells comprised between 15% and 50% of the bulk tumor cell population in multiple independent mammary tumors from three different transgenic mouse models of breast cancer. Culture of primary mammary tumor cells in chemically-defined, serum-free medium as non-adherent tumorspheres preserved TIC frequency to levels similar to that of the primary tumors from which they were established. By contrast, propagating the primary tumor cells in serum-containing medium as adherent populations resulted in a several thousand-fold reduction in their tumor-initiating cell fraction. Our findings suggest that experimental conditions, including the sensitivity of the transplantation assay, can dramatically affect estimates of tumor initiating cell frequency. Moreover, conditional on cell culture conditions, the tumor-initiating cell fraction of bulk mouse mammary tumor cell preparations can either be maintained at high or low frequency *in vitro* thus permitting comparative studies of tumorigenic and non-tumorigenic cancer cells.

## Introduction

Tumor-initiating cells (TICs), often termed cancer stem cells, are functionally defined by their capacity to re-grow a new tumor after transplant into experimental animals that recapitulates the phenotype of the primary tumor from which the cells were isolated, and which can be serially transplanted thus demonstrating their capacity to differentiate and self-renew [1]. TICs were first identified in acute myelogenous leukemia [2], and thereafter in many solid tumors [3]–[7] including those of the breast [8]. TICs and tissue-specific adult stem cells share phenotypic and functional properties leading to the suggestion that they originate from adult stem cells or from progenitor cells that acquire stem cell traits [9]–[11]. TICs are infrequent in most human tumors, rarely exceeding 0.01% of the bulk tumor cell population [3]–[6], [8], [12], [13]. However, recent findings in mouse cancer models [14]–[20] and human melanomas [21] demonstrate that TIC frequencies can approach 25% of the bulk tumor cell population calling into question the generality of the cancer stem cell model. However, various parameters influence TIC frequency in bulk tumor cell preparations including the methods used to isolate and process tumor samples, the site of tumor cell injection, the extent of the immune-deficiency of the recipient host, the duration of the observational period following tumor cell transplant, and whether agents that facilitate tumor cell engraftment such as Matrigel or stromal cells are co-injected with the tumor cells [21]. Hence the frequency of TICs in tumors is insufficient to distinguish malignancies that follow the cancer stem cell model from those that do not.

Studies of human breast TICs are challenging for a number of reasons. Breast tumors are generally small at the time of resection thus providing relatively few bulk tumor cells for TIC purification and analyses [8]. Moreover, current cell purification methods yield TIC preparations that at best comprise ∼1–2% of the total tumor cell population thus compromising their specific study [8], [22]. To overcome these limitations we investigated whether mammary tumors of transgenic mice might afford a more plentiful and renewable source of TICs for investigation. Whereas the available mouse models of breast cancer do not wholly reproduce the diversity of human breast tumor subtypes, in part because most mouse mammary tumors rarely express the estrogen receptor, morphological analyses [23], [24], biomarker studies [25] and global transcript profiling [26] suggests that they provide approximate replicas of their human subtype counterparts. For example, mammary tumors occurring in the Neu and polyomavirus middle tumor antigen (mT) models are morphologically similar to certain human breast tumor histological subtypes [24], [25], and share a gene expression signature characteristic of the luminal molecular subclass of human breast tumors [26]. Similarly mouse mammary tumors resulting from overexpression of Wnt/beta (β) – catenin pathway components mimic those of the basal-like molecular subtype of human breast tumors [26].

Mouse mammary TICs have been identified in p53-null mice and in transgenic mice genetically engineered to overexpress Wnt-1 or Neu (Erbb2 or Her-2) in their mammary epithelium [17]–[20]. The TIC frequency among tumors from each of the models varies between 0.01%–1% of the bulk tumor cell population. Whereas the frequency of TICs in different tumors arising in the same model as determined by an individual research group is roughly equivalent, different laboratories report up to 50-fold differences in TIC frequencies in tumors arising in the same model [17]–[20], [27]. For example, the TIC frequency reported by Vaillant et al in tumors of a Wnt-1 transgenic strain (0.56%) supersedes that reported by Cho et al (0.013%) by over 40 fold [17], [19].

We employed several mouse models of human breast cancer, where the mouse mammary tumor virus (MMTV) promoter directs transcription of the rat *neu* proto-oncogene [28] or oncogenes encoding either mT [29] or a stable mutant form of β-catenin in mammary epithelial cells to determine their tumor-resident TIC frequency and to learn whether this parameter differed among the models. The tumors arising in the transgenic strains display characteristic histopathologies, vary in their cellular composition and have been inferred to originate from the oncogenic transformation of mammary epithelial stem cells or particular progenitor cells [30], [31]. Neu-induced tumors comprise a relatively homogenous tumor cell population that primarily expresses luminal-lineage markers [24], whereas tumors induced by expression of mT or by activation of the Wnt/β-catenin pathway are heterogeneous comprising both luminal- and myoepithelial-lineage-biased cells [8], [24], [30], [32]. The extent of the cellular heterogeneity of Wnt-1 induced tumors varies among individual tumors [24] and that of the mT-generated tumors increases during their progression [25].

We determined the TIC fraction in multiple mammary tumors arising in each of the models and found that, independent of the model, all the tumors comprised between 15% and 50% TICs. Culture of primary tumor cells from tumors of the MMTV-Neu model in serum-free medium, conducive for stem cell self-renewal [33], led to the formation of non-adherent spheres, termed tumorspheres [34], that comprised a TIC frequency similar to that of the tumor from which the cells were isolated. By contrast, when the primary tumor cells were placed in serum-containing medium, conditions that initiate a differentiation program in human and mouse mammary epithelial stem/progenitor cells [35], [36], [37], [38], the tumor cells adhered to the substratum and proliferated resulting in the net expansion of the tumor cell population, but the TIC frequency declined by multiple orders of magnitude compared to that in bulk primary tumor cells. Hence, the mouse mammary tumors we have investigated provide an abundant and renewable tumor cell populations, which can be manipulated *in vitro* to derive TIC-rich or -poor cell populations for comparative studies.

## Materials and Methods

### Care and treatment of mice

All mice used in these experiments were housed in an Canadian Council on Animal Care (CCAC)-approved facility at McMaster University. Mice were provided with food and water *ad libitum*. All animal experiments were approved by the McMaster University Animal Research Ethics Board (AUP: 10-01-04).

### Isolation and transplant of primary tumor cells

We have reported the methods used to isolate, process and transplant mammary epithelial cells and tumor cells [38], [39]. In short, tumors were surgically removed from anesthetized transgenic female mice and minced with a scalpel in Versene. Roswell Park Memorial Institute (RPMI) medium containing trypsin (1 mg/ml) and collagenase A (3 mg/ml) and 2% fetal bovine serum (FBS) were added and the tissue fragments incubated for 15 minutes at 37°C. The tumor cell suspension was titruated by repeated pipetting with a 5 ml pipette and incubated for another 15-minute period at 37°C. The tumor cell suspension was titruated again at the end of the second incubation period and the dissociated cells were filtered through 40 µm cell strainers (Falcon; Franklin Lakes, NJ USA) and concentrated by centrifugation at 1,500 rpm for 15 minutes at room temperature. Cells were washed in 50 ml volumes of Ham's F12 (Invitrogen; Carlsbad CA, USA) until the supernatant was clear of red blood cells. The cells were stained with trypan blue and the fraction of viable cells calculated using a hemocytometer. Approximately 90–95% of the cells prepared from tumors were viable as visualized microscopically. Cells to be injected into the “cleared” number 4 mammary fat pad were suspended in 50% Matrigel (BD Biosciences; Bedford MA, USA) (in phosphate-buffered saline [PBS] supplemented with 5% FBS and 0.5% trypan blue). Ten microliters of the Matrigel/cell suspension were injected into the cleared fat pads of recipient females. When single cells were injected, the cells were diluted to 0.1 cell/microliter and the occurrence of a single cell in 10 microliters of this solution deposited in a Terasaki plate was confirmed microscopically before injecting the sample. Mice transplanted with tumor cells were examined for tumors at either 4 or 16 weeks post cell transplantation and were scored positive if any tumor-like masses were observed. TIC frequencies for single-cell seeded tumors were determined using ELDA software (http://wehi.edu.au/software/elda/index.html [40], whereas TIC frequency of the adherent tumor cell population was estimated using L-calc Software (Stem Cell Technologies; Vancouver BC, Canada).

### Whole mount analyses

Mammary fat pads from recipient mice were spread onto glass slides, allowed to air-dry and subsequently stained in Harris Haematoxylin (Fisher Scientific; Fair Lawn NJ, USA). Excess stain was removed from the whole mounts by washes in 70% ethanol and 1% hydrochloric acid, and were dehydrated in 100% ethanol, cleared in xylenes and mounted on glass slides with Permount (Fisher Scientific). Stained whole mounts were examined on a Leica Diaplan (Leica; Concord ON, Canada) dissecting microscope and photographed.

### Histology

Tumors were fixed in 4% paraformaldehyde (EM Science; Gibbstown NJ, USA) for 24 hours at room temperature, embedded in paraffin, cut into 4 µm sections and stained with Harris Haematoxylin and Eosin (H&E) solution. All images were captured using a Leica inverted microscope (Leica) and Open Lab Improvision software (Perkin Elmer; San Jose CA, USA).

#### Immunoflorescent staining

The expression of mammary epithelial cell lineage markers in tumor sections was determined as described previously [38]. In brief, antigen retrieval was performed on tumor sections using Antigen Unmasking solution (Vector Labs; Burlingame CA, USA) prior to blocking in 3% normal goat serum (Dako Cytomations; Carpinteria CA, USA). All antibody incubations were performed for 1 hour at room temperature. The primary antibodies included those that bind to alpha-smooth muscle actin (α-Sma) (mouse monoclonal, Sigma; Saint Louis MO, USA), cytokeratin (CK) 14 (rabbit polyclonal, Covance; Emeryville CA, USA), and CK8 (rat monoclonal, Developmental Studies Hybridoma Bank; Iowa City IA, 52242 USA). Alexa Fluor – 488 or Alexa Fluor- 594 conjugated secondary antibodies were used in conjunction with the goat anti-rat and goat anti-rabbit primary antibodies. The Alexa Fluor – 488, signal-amplification kit (Molecular Probes, Eugene OR, USA) was used to detect the primary mouse monoclonal antibody bound to α-Sma (Sigma). Slides were prepared with Vecta-shield mounting media containing 4′, 6′- diamindino-2-phenylindole (DAPI) (Vector Laboratories) to visualize nuclei.

### Culture of tumor cells in vitro

Freshly isolated primary tumor cells were placed in serum-free medium comprising Dulbecco Modified Eagle Medium (DMEM): Ham's F-12 (3∶1), 4 μg/ml of B-27, 20 ng/ml Epidermal Growth Factor (EGF), 40 ng/ml Fibroblast Growth Factor-2 (FGF-2) and 4 ng/ml Heparin, and plated into ordinary T25 tissue culture flasks (all from Invitrogen) to generate tumorspheres [33]. The primary tumorspheres that arose 4 days later were mechanically dissociated (titruation) to derive a single cell suspension and the dispersed cells plated in serum-free medium containing the growth factors described above at a density of ∼30,000 cells per ml; this process was repeated for a total of 3 serial passages [38]. The cells were dissociated by titruation from the serially-passaged tumorspheres prior to their injection into mice as described above. Dispersed primary tumor cells were similarly plated into T25 flasks in serum-containing medium (DMEM containing 10% FBS). The adherent cells were epithelial in morphology suggesting they originated from tumor epithelial cells; fibroblastic cells were not apparent in these cultures. The tumor cells were removed from the plastic surface using trypsin (0.25% in Versene) at 37°C, diluted in serum-containing medium and plated as described above. The adherent tumor cells were passaged every 4 days for 3 passages before measuring their TIC frequency as described above. Use of different serum lots did not affect the TIC frequency of the adherent cultures.

## Results

**Figure 1:**
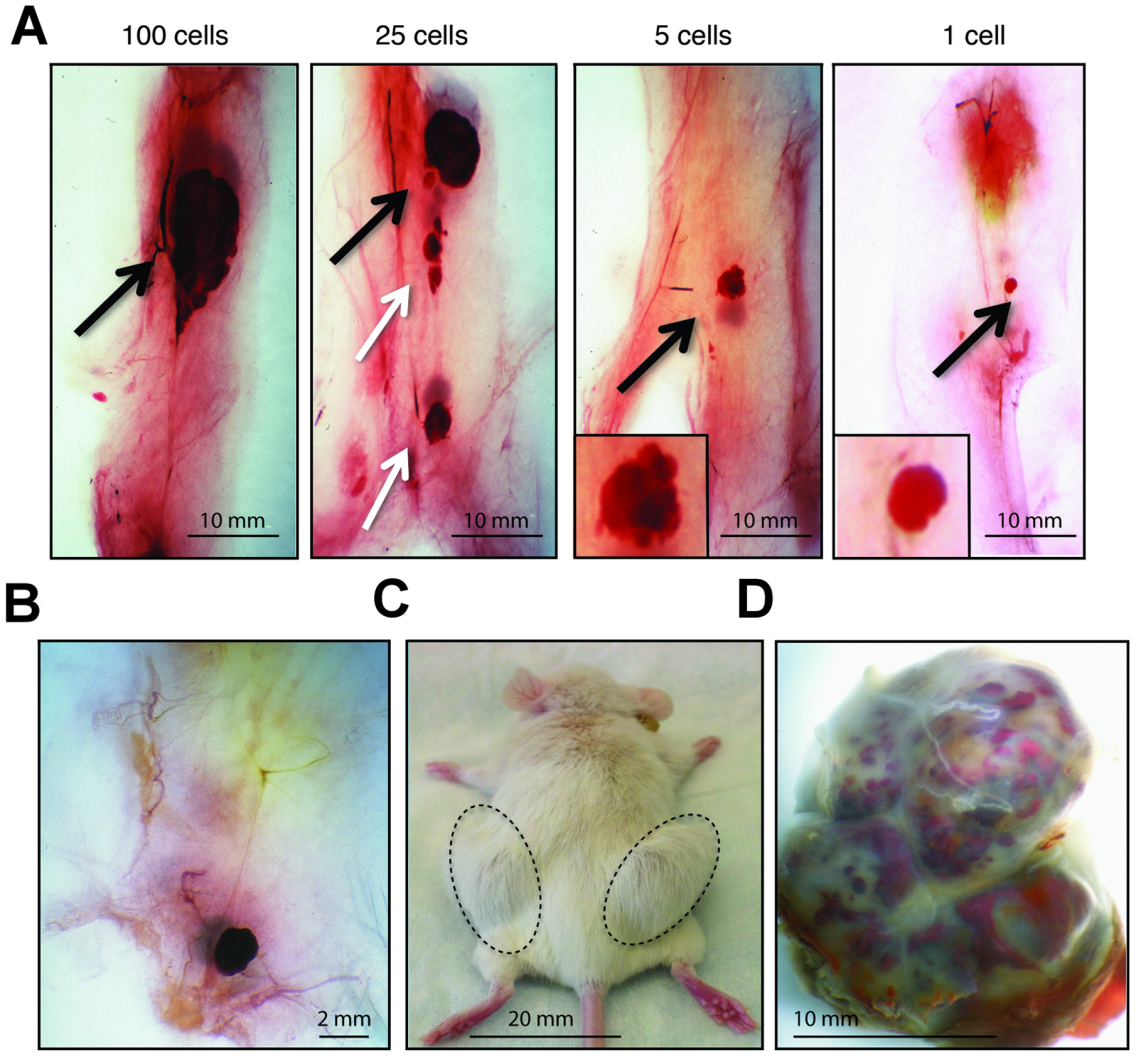
Limiting dilution transplants of dispersed primary MMTV-Neu transgenic mammary tumor cells. (**A**), Haematoxylin-stained mammary whole mounts isolated 4 weeks post-transplant reveal tumor-like masses in the fat pads of recipient mice injected with limiting dilutions of primary tumor cells. The mass of the tumor nodules correlates with the number of tumor cells injected (black arrows). Satellite tumor nodules are apparent along the needle track (white arrows). The images were photographed at a magnification of 6.4. (**B**), Tumor nodule appearing in mice transplanted with a single cell at 4 weeks post-transplantation. The image was photographed at a magnification of 6.4. (**C**), Macroscopic, palpable tumors are visible 16 weeks after transplanting single cells into the #4 mammary fat pads of a recipient mouse. (**D**), Single-cell induced tumors can develop into macroscopic tumors constituting over 10 million cells.

Initial limiting dilution tumor cell transplant experiments were carried out with mammary tumors from transgenic mice of the MMTV-Neu (N202) strain that express the wild type rat Neu cDNA in mammary epithelial cells [28]. Tumors arise in this model after long latency (∼315 days in 50% of virgin female mice) [39] due in part to deletions in the Neu cDNA encoding the juxta-membrance region of the protein leading to constitutive activation of its tyrosine kinase activity [41], [42]. To estimate the frequency of TICs in these tumors, limiting dilutions of dispersed primary tumor cells were transplanted into the mammary fat pads of syngeneic FVB/N mice. The mice were sacrificed 4 weeks later, at a time when palpable tumors had developed in animals injected with 10^4^ or 10^5^ tumor cells. Small tumor-like masses, which we termed tumor nodules, were found near the cell injection site, corresponding in size to the number of transplanted tumor cells (Fig. 1 A, black arrows). Unexpectedly, tumor nodules were found in mice transplanted with single cells. The masses resulting from transplant of between 5 and 100 tumor cells appeared to be a collection of individual nodules resembling those that had formed in fat pads injected with a single cell. Nodules approximating the size of those seeded by single cells were often observed along the needle track (Fig. 1A, white arrows). These findings suggested that single transplanted tumor cells proliferated in the mammary fat pads of their host thereby forming nodules, and that transplant of multiple tumor cells into individual fat pads yielded aggregates of multiple nodules.

To learn whether the tumor nodules appearing in mice transplanted with single cells were indeed nascent tumors and could form *bona fide* tumors, we repeated the transplants with cells from a different mammary tumor and sacrificed the recipient hosts 4 or 16 weeks thereafter. Single tumor cells, visualized microscopically prior to transplant, were injected into syngeneic female mice. Tumor nodules were found in many of the recipients 4 weeks after cell transplantation as observed previously (Fig. 1B), whereas large tumors were apparent 16 weeks post cell transplantation (Fig. 1 C and D). These findings suggest that the tumor nodules observed 4 weeks post-transplantation progress to form large tumors 16 weeks after the initial transplant of single primary tumor cells.

To determine the frequency of TIC in individual mammary tumors and to learn whether this fraction varied among the tumors, we performed limiting dilution cell transplants with tumor cells from additional independent Neu-induced tumors. Tumors were present 16 weeks post transplant in 20 of 40 host mice inoculated with single tumor cells from each of 6 independent Neu-induced mammary tumors (Table 1). The TIC frequency calculated from single cell transplants ranged from 1/2–1/6 (95% confidence interval [95% CI]) [43]. Limiting dilution cell transplantation experiments were also performed with 2 mammary tumors that arose in mT transgenic mice and 3 that formed in the stable β-catenin transgenic strain. The TIC fraction in these tumors averaged 1/3 (1/2–1/6; 95% CI) for the mT model and 1/3 (1/2–1/8; 95% CI) for the stable β catenin model.

**Table 1:** Tumor-initiating cell frequencies in primary tumors.

*MMTV-Transgene*	*Tumor Identifier*	*Number of Primary Tumor Cells Transplanted*	*Fraction of Primary Tumor Cells Engrafted*	*TIC Frequency (95% CI)*
Neu	3740	100	2/4	1/6
		25	1/4	(1/40–1/2)
		5	2/6	
		1	1/6	
Neu	3314	100	1/2	1/2
		25	4/4	(1/6–1/2)
		5	4/6	
		1	3/6	
Neu	3691	100	4/4	1/6
		25	4/4	(1/40–1/2)
		5	6/6	
		1	1/6	
Neu	3727	100	4/4	
		25	4/4	1/2
		5	2/6	(1/4–1/2)
		1	4/6	
Neu	3738	100	2/4	
		25	2/2	1/2
		5	4/6	(1/3–1/2)
		1	6/8	
Neu	3852	100	3/4	
		25	3/4	1/3
		5	1/6	(1/8–1/2)
		1	5/8	

**Figure 2:**
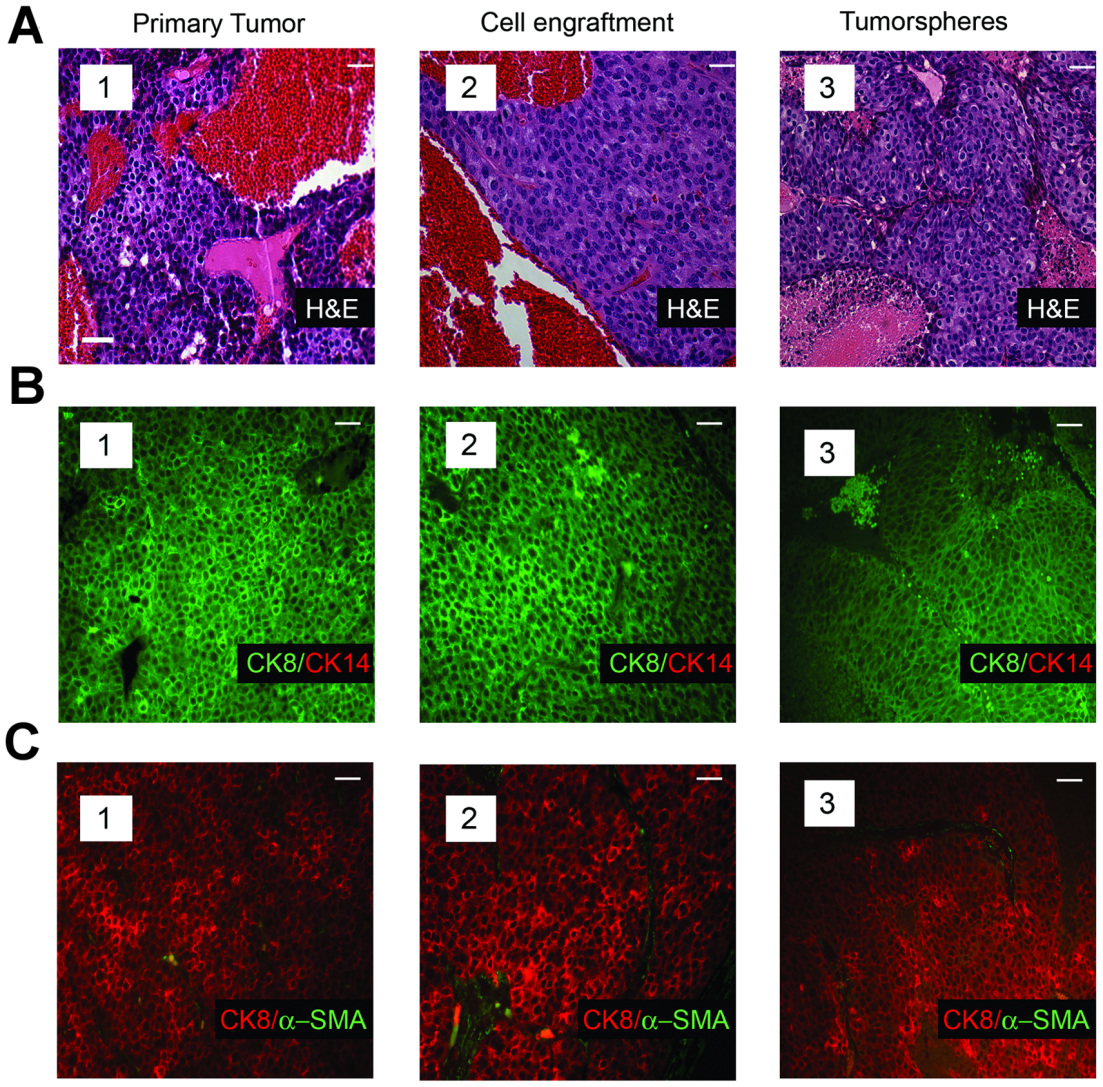
Tumors arising from transplant of single tumor cells recapitulate the histology and cellular composition characteristic of the parental tumors. (**A**), Histopathology of a primary mammary tumor from an MMTV-Neu transgenic mouse (subpanel 1), that of a tumor seeded by transplant of a single cell (subpanel 2) from the same primary tumor, and that of a tumor derived from transplant of a single cell dissociated from tumorspheres of the same transgenic strain (subpanel 3). H&E staining of tumor sections illustrates the solid and nodular cytoarchitecture characteristic of Neu-induced tumors. (**B**), Immunofluorescence analyses of an MMTV-Neu tumor (subpanel 1), that of a tumor seeded by transplant of a single cell (subpanel 2) from the same primary tumor, and that of a tumor (subpanel 3) seeded by transplant of a single tumorsphere-derived cell stained with antibodies to CK8 and CK14. (**C**), Immunofluorescence analyses of an MMTV-Neu tumor (subpanel 1), that of a tumor seeded by transplant of a single cell (subpanel 2) from the same primary tumor, and that of a tumor (subpanel 3) seeded by transplant of a single tumorsphere-derived cell stained with antibodies to CK8 and α-SMA. Note that distinct sections of the same tumor were used for the analyses shown in panels a–c. Scale bar (inset) represents 40 µm in all panels.

**Figure 3:**
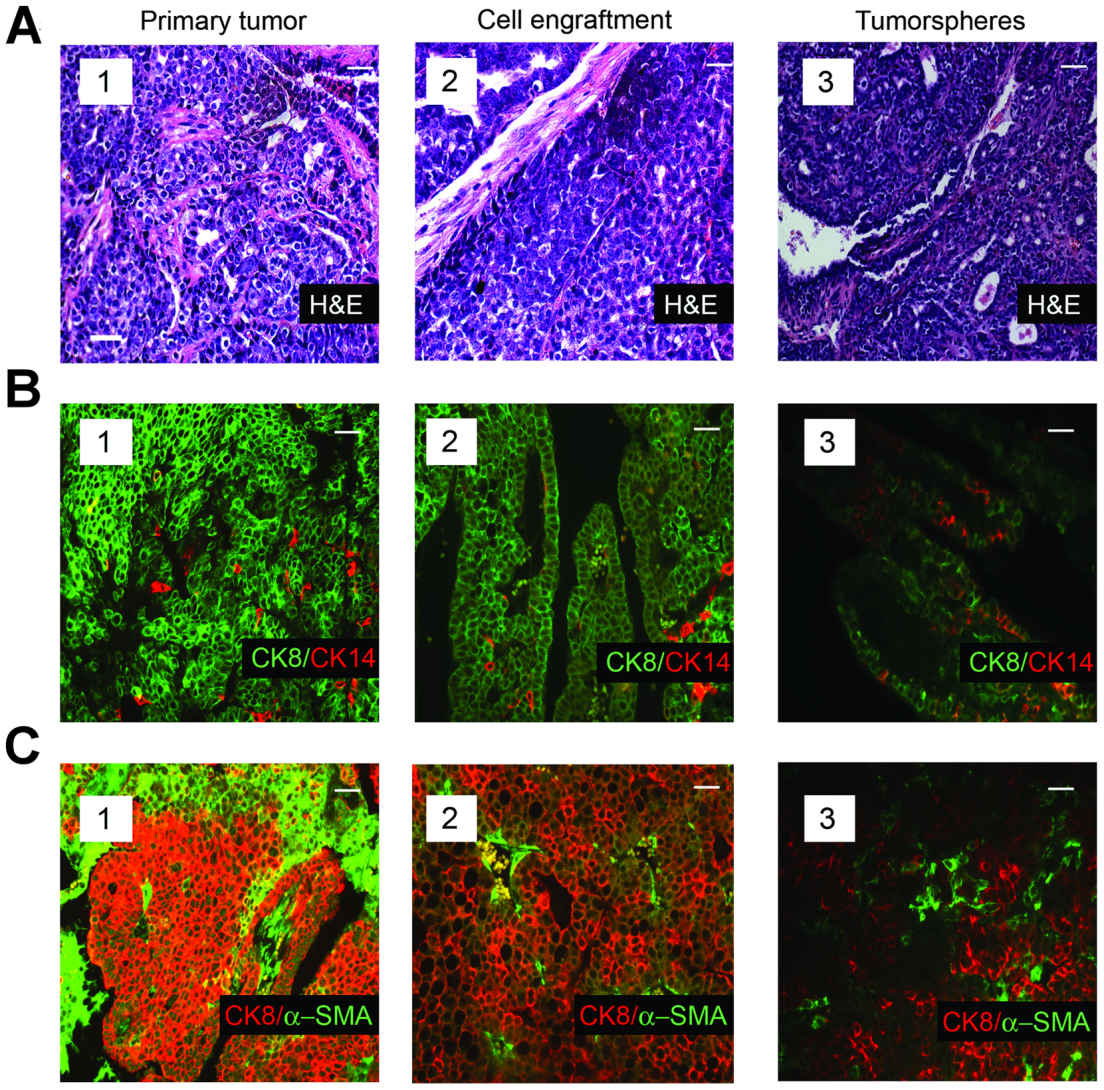
Single transplanted tumor cells isolated from the mammary tumors of MMTV-mT transgenic mice recapitulate the histology and cellular composition characteristic of their parental tumors. (**A**), Histology of a primary MMTV-mT tumor (subpanel 1), that of a tumor resulting from transplanting a single tumor cell from the same tumor (subpanel 2), and that of a tumor that arose from transplantating a single tumorsphere-derived cell from a tumor of the same transgenic strain (subpanel 3). (**B**), Immunofluorescence analyses of an MMTV-mT tumor (subpanel 1), that of a tumor seeded by transplant of a single cell from the same primary tumor (subpanel 2), and that from a tumor resulting from transplant of a single tumorsphere-derived cell from a tumor of the same transgenic strain stained with antibodies to CK8 and CK14 (subpanel 3). (**C**), Immunofluorescence analyses of an MMTV-mT tumor (subpanel 1), that of a tumor seeded by transplant of a single cell from the same primary tumor (subpanel 2), and that of a tumor resulting from transplant of a single tumorsphere-derived cell from a tumor of the same transgenic strain stained with antibodies to CK8 and α-SMA (subpanel 3). Note that distinct sections of the same tumor were used for the analyses shown in panels a–c. Scale bar (inset) represents 40 µm in all panels.

**Figure 4:**
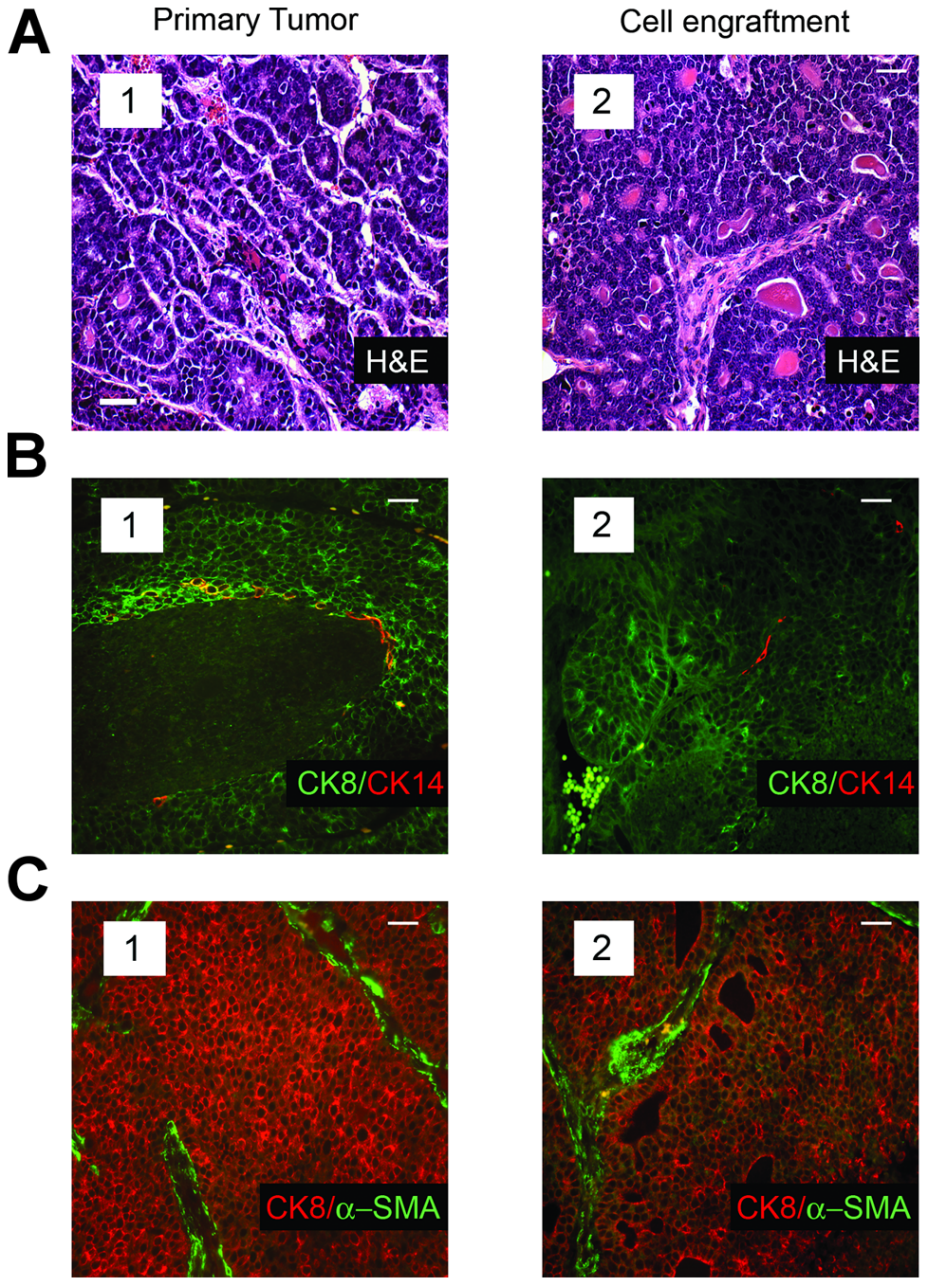
Single transplanted tumor cells isolated from the mammary tumors of MMTV-β-catenin transgenic mice recapitulate the histology and cellular composition characteristic of their parental tumors. (**A**), H&E staining of an MMTV-β-catenin primary tumor (subpanel 1) and a tumor obtained after transplanting a single tumor cell originating from the same tumor (subpanel 2). (**B**), Immunofluorescence analyses of an MMTV-β-catenin tumor (subpanel 1) and that of a tumor seeded by transplant of a single cell from the same primary tumor stained with antibodies to CK8 and CK14 (subpanel 2). (**C**), Immunofluorescence analyses of an MMTV-β-catenin tumor (subpanel 1) and that of a tumor seeded by transplant of a single cell from the same primary tumor stained with antibodies to CK8 and α-SMA (subpanel 2). Note that distinct sections of the same tumor were used for the analyses shown in panels a–c. Scale bar (inset) represents 40 µm in all panels.

To learn whether tumors arising after single cell transplants recapitulated the phenotype of the tumor from which they were derived, we compared the histology and cellular composition of the tumors in the transgenic strains with those occurring in the mice transplanted with genetically matched individual tumor cells from the same parent tumors. Antibodies to a luminal-lineage marker (CK8) and those to each of two myoepithelial-lineage markers (CK14 and α-SMA) were used to characterize the cellular composition of the tumors. The morphology and cellular composition of the mammary tumors in the Neu (Fig. 2 A–C subpanel 1) and mT (Fig. 3 A–C subpanel 1) transgenic mice was similar to that reported previously [24], [25], [44]. The tumors arising from transplants of single tumor cells isolated from tumors occurring in each model (Neu and mT) reproduced the histopathology (Fig. 2A subpanel 2, and Fig. 3A subpanel 2) of the corresponding parental tumors. Similarly the cellular composition of the tumors originating in mice transplanted with single Neu-tumor cells (Fig. 2B subpanel 2 and Fig. 2C subpanel 2), or single mT-tumor cells (Fig. 3B subpanel 2 and Fig. 3C subpanel 2) reproduced that of the corresponding parental tumors. Whereas the mammary tumors in mice resulting from activation of the Wnt/β-catenin pathway span a range of morphologies and vary in their proportion of luminal-lineage and myoepithelial-lineage restricted cells, the tumor-specific characteristics of each primary tumor was reproduced in secondary tumors seeded by individual tumor cells that had been isolated from the same primary tumor (Fig. 4, compare panels 1 and 2).

To determine whether the tumors resulting from single cell transplants comprised self-renewing TIC, we performed serial transplants of the engrafted tumors [45], [46]. Tissue fragments (∼1 mm^3^) comprising ∼5,000 tumor cells from the single cell-seeded tumors from each of the three models formed secondary tumor grafts in over 80% of transplanted hosts, which could be similarly serially transplanted for up to 10 successive passages before this experiment was discontinued (data not shown). Hence the tumors of all three breast-cancer prone mouse models comprised TIC capable of recapitulating the phenotype of the primary tumors from which they were isolated and were capable of self-renewal, even when the tumors originated from transplant of single tumor cells.

High TIC frequency, lack of an apparent cellular hierarchy and the inability to prospectively enrich TIC from bulk tumor cell populations using antibodies to a diversity of cell-surface proteins are features that have been ascribed to tumor types that do not follow the cancer stem cell model [47]. The mouse mammary tumors we analyzed encompass some of these aforementioned characteristics (viz. high TIC frequency), but also possess features that are commonly attributed to tumors that follow the cancer stem cell model (viz. cellular hierarchy). Hence we wondered whether TIC frequency could be manipulated by propagating TIC-rich primary tumor cells under conditions that either facilitate stem cell self-renewal or differentiation.

**Figure 5:**
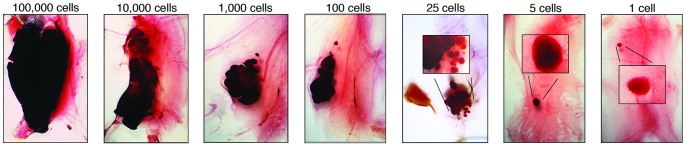
Single tumorsphere-derived cells from tumors of the MMTV-Neu strain seed the growth of tumors after transplantation into syngeneic FVB/N mice. Haematoxylin-stained mammary whole mounts isolated 4 weeks post-transplant of varying numbers of tumorsphere-derived cells reveal tumor-like masses in the fat pads of recipient mice. The mass of the tumor nodules directly correlated with the number of injected tumor cells. The images were photographed at a magnification of 6.4.

The cancer stem cell model predicts that experimental conditions facilitating stem cell self-renewal will increase or maintain TIC frequency, whereas those stimulating differentiation will reduce their frequency. Consequently, we propagated freshly-isolated primary tumor cells in either serum-free medium, which facilitates the survival and self-renewal of human or mouse mammary epithelial stem/progenitor cells as non-adherent mammospheres [37], [38], or in serum-containing medium, which stimulates the differentiation of mouse [38], [48] and human mammary epithelial progenitor cells [36]. We previously reported that primary tumor cells from tumors of the mouse models form non-adherent spheres, which we termed tumorspheres [34]. A limiting dilution cell transplantation experiment was performed with cells dissociated from tumorspheres established from a single tumor. Tumors or tumor nodules appeared in nearly all the mice implanted with tumorsphere-derived cells including one of four mice transplanted with a single tumor cell (Fig. 5; Table 2 – Tumor identifier 3,736). The latter findings were reminiscent of the data obtained using primary tumor cells (Fig. 1).

**Table 2:** Tumor-initiating cell frequencies in tumorpshere preparations.

*MMTV-Transgene*	*Tumor Identifier*	*Number of Tumorsphere-Derived Cells Transplanted*	*Fraction of Tumorsphere-Derived Cells Engrafted*	*TIC Frequency (95% CI)*
Neu	3736	100,000	4/4	
		10,000	4/4	
		1,000	6/6	
		500	4/4	
		100	12/12	1/3
		50	4/4	(1/4–1/2)
		25	2/2	
		10	3/4	
		5	4/4	
		1	12/28	
Neu	3727	100	4/4	
		25	4/4	1/5
		5	6/6	(1/10–1/3)
		1	6/28	
Neu	3738	25	3/4	1/3
		5	4/6	(1/8–1/2)
		1	3/8	
Neu	3852	25	3/4	1/3
		5	3/8	(1/8–1/2)
		1	3/8	

We also compared the frequency of TIC in three additional independent mammary tumors with that in tumorspheres derived from these same tumors. The tumorspheres were passaged 3–4 times during a 2–3 week period to limit any potential genetic changes in the sphere-resident cells that might alter TIC frequency. Mice transplanted with dilutions of the dispersed tumorsphere-derived cells were sacrificed 2–4 months after cell transplantation. Table 2 illustrates that the frequency of TIC in the tumorspheres was comparable to that in the primary tumor cell preparations from which they were derived (compare TIC frequency in primary tumors 3,727; 3,738; and 3,852 [Table 1] with companion tumorspheres derived from these tumors [Table 2]). Moreover, the histology (Fig. 2A, compare subpanels A1 and A3) and cellular composition of the tumors seeded by single tumorsphere-derived cells were very similar to those of their parental tumors arising in the corresponding transgenic strains (Fig. 2 B and C, compare panels B1 and B3, and C1 and C3). Individual tumorsphere-derived cells established from primary tumors of the mT model similarly recapitulated the histology (Fig. 3A, compare subpanels a1 and a3) and cellular composition of the primary tumors (Fig. 3B, compare subpanels B1 and B3, and C1 and C3). Hence culture of primary tumor cells as non-adherent spheres maintained a high TIC frequency similar to that of the tumors from which the cells were isolated, and transplant of tumorsphere-derived cells into syngeneic mice as single cells yielded tumors that phenocopied the primary tumors from which they had been established.

To assess any effect of culturing primary tumor cells in serum-containing medium on TIC frequency, we propagated freshly isolated primary tumor cells from 3 independent tumors that occurred in the MMTV-Neu strain in this medium. In pilot experiments we found that the primary tumor cells adhered and proliferated resulting in a net expansion of the cell population for 4–6 serial passages, but the total tumor cell population subsequently declined with each successive passage and these cultures could not be sustained beyond 7–9 serial passages. In consequence, we limited propagation of the primary tumor cells to 3 serial passages at 4-day intervals, a protocol similar to that used to propagate tumorspheres. We observed that independent of the tumor source between 10,000 and 100,000 tumor cells that had been propagated in serum-containing medium were required to elicit a new tumor after transplant into syngeneic female mice (Table 3 – Tumor identifiers 001, 002 and 406). The tumors seeded by the cells propagated in serum-containing medium reproduced the histology of the primary tumors from which the cells originated (Fig. S1, compare A and B) suggesting that infrequent TIC in these tumor cell populations seeded tumor growth. Hence *in vitro* culture of TIC-rich primary tumor cells in serum-containing medium for a relatively short period drastically reduced TIC frequency during a time when the tumor cell population expanded.

**Table 3:** Tumor-initiating cell frequencies in adherent tumor cell preparations.

*MMTV-Transgene*	*Tumor Identifier*	*Number of Adherent Tumor Cells Transplanted*	*Fraction of adherent tumor cells engrafted*	*TIC Frequency (95% CI)*
Neu	001	100,000	1/4	1/392,318 (1/2,765831–1/55,648)
		10,000	0/4	
		1,000	0/4	
		100	0/4	
		10	0/4	
Neu	002	50,000	4/4	1/20,332
		10,000	1/4	(1/54,184
		1,000	0/4	-
		100	0/4	1/7,629)
		10	0/4	
Neu	406	10,000	0/4	N/A
		1,000	0/4	
		100	0/4	
		10	0/4	

## Discussion

The TIC frequency in minimally manipulated bulk tumor cell populations from tumors occurring in 6 distinct mouse models of breast cancer have now been determined. The latter models include: transgenic mice engineered to overexpress Wnt-1 [17], [19] the Neu proto-oncogene [19] or a mutant constitutively-active form of Neu [20] all under the control of the MMTV promoter; p53-null mice [18], [19], [27]; as well as the MMTV-mT and MMTV-beta-catenin models reported in this study. TIC frequencies in the MMTV-Wnt-1 model as determined by two different research groups varied by more than 40 fold [17], [19]. TIC frequencies in two related Neu models similarly varied by 30 fold – between 0.03% (MMTV-mutant Neu) [20] and 0.9% (MMTV-proto-Neu) [19]. Indeed TIC frequencies reported by the same group in two different studies published years apart using the p53-null model varied by 13 fold [18], [27].

The TIC frequencies reported previously did not exceed 1% of the bulk tumor cell population in any of the mouse models and generally were much lower than this value [17]–[19], [27]. Our measures of TIC frequency in tumors of the three models that we examined averaged 30%. The TIC fraction in the tumors arising in the MMTV-Neu (N202) and MMTV-β-catenin transgenic strains substantially exceeds those reported previously in the same (MMTV-Neu) [19] or related (MMTV-Wnt-1) [17], [19] models by 30 fold or more. Indeed unpurified single tumor cells from tumors of each of the three models we examined initiated tumor growth at high efficiency after orthotopic transplant into immune-competent syngeneic mice demonstrating that TIC autonomously initiate tumor growth and implying that TIC must be therapeutically targeted to achieve durable breast cancer remission. The single cell seeded tumors recapitulated the cellular composition of the tumors from which they were isolated and these engrafted tumors could be serially transplanted demonstrating their capacity for differentiation and self-renewal. The high TIC frequency in the mouse models we analyzed may be due to the high expression of the various oncogenes under the control of the MMTV promoter. High oncogene expression has previously been shown to expand the repertoire of hematopoietic target cells that are transformed by *MLL-AF9* [49]. The MMTV promoter may similarly effect high expression of various oncogenes to mammary epithelial cell types that are ordinarily not susceptible to transformation by these oncogenes when expressed at lower levels.

The wide discrepancies in TIC frequency in tumors of the same models reported by various investigators and us likely result from differences in the experimental procedures used to prepare and fractionate dispersed tumor cells, and perhaps to the sensitivity of the tumor cell transplantation assay as practiced in individual laboratories. Unlike previous measurements of TIC frequency in mouse mammary tumors we did not use fluorescence activated cell sorting (FACS) to separate hematopoietic, endothelial and stromal cells from tumor cells prior to their transplantation, whereas all previous studies employed FACS to enrich for tumor cells [19], [27], [43]. The high pressure of FACS may reduce TIC viability as reported previously [27], [43]. Moreover TIC engraftment may be aided by non-tumor derived cells such as stromal cells [50]; the depletion of such cells from the bulk tumor cell population in other studies may have led to underestimates of TIC frequency.

We used Matrigel as a vehicle to enhance TIC engraftment, whereas none of the other studies used this agent. Matrigel reportedly facilitates the engraftment of mouse mammary epithelial stem and progenitor cells and may have similarly stimulated the engraftment of TIC and progenitor-like tumor cells in our transplantation experiments [51]. In this regard we observed both large (1–2 g wet weight) and small tumors (100–200 mg wet weight) among recipient mice transplanted with limiting dilutions of primary tumor cells. However, the small tumors, which may have been seeded by progenitor-like tumor cells with limited proliferative potential, constituted only a minor fraction of all the tumors we identified and were not scored in our analyses of TIC frequency or analyzed further.

The procedure we used to prepare dispersed tumor cells differed from those of previous studies; indeed none of the published studies used the same conditions to dissociate tumor cells from tumor tissue. Previous work employed only collagenase [18], collagenase and hyaluronidase [20], and collagenase, hyaluronidase and trypsin [19]. The duration of treatment of minced tumor tissue with these enzymes also varied ranging between 1 and 5 hours. We used both collagenase and trypsin to dissociate cells from tumor fragments and incubated the tissue for a period of 30 min following a protocol by Pullan and Streuli [52].

It is conceivable that the conditions we used to prepare tumor cells may have led to the selective recovery of TICs after filtering the bulk tumor cells through a 40 µm sieve to remove undigested tumor fragments and tumor cell aggregates. The yield of tumor cells from a 1–2 gram tumor arising in the MMTV-Neu (N202) strain approximated ∼ 2–3×10^8^ cells prior to the removal of tumor cell aggregates by filtration through a 40 µm sieve; ∼95% of these cells were viable. After filtration through the sieve ∼20% of the cells were recovered in the filtrate; ∼95% of these cells were viable. The latter cell yield is in keeping with that reported previously from the mammary glands of mice using the Pullan and Streuli protocol [52]. If the frequency of TIC in the Neu-induced tumors was as high as 1% as reported previously [19], then we would expect a 5-fold enrichment in the TIC fraction in the filtrate yielding a ∼5% TIC frequency. However, our measurements yielded a 6 fold higher TIC frequency in the filtrate, which averaged ∼30%. We also note that the mouse mammary tumorspheres were dissociated by titruation, and were not filtered through sieves, yet their resident TIC frequency approximated that of the primary tumors from which they were established. Thus whereas we cannot rule out the possibility that the means we used to isolate dispersed tumor cells enriched for TIC, we doubt that the high TIC frequencies we observed in the tumors we examined was due solely to this factor. It is also conceivable that the protocols used to dissociate the tumors, the quality of the enzymes employed in each study, and the duration of exposure of tumor tissue to these reagents used in previous studies may have led to the selective loss of TIC and may account for the striking differences in TIC frequencies reported by others and us. Resolution of the latter will require further study.

It is also not clear whether the extent of progression of the tumors analyzed in the various studies influenced TIC frequency. Multiple different TICs may be present during the early stages of tumorigenesis, but clonal evolution may occur during tumor progression resulting in the selection of a TIC population with an increased propensity to self-renew and a decreased capacity to differentiate [47], [53]. The mammary tumors we investigated may have progressed to a greater extent than those studied by others and this parameter may account for the higher TIC fraction that we observed. Evidence for a relationship between tumor progression and TIC frequency is suggested by recent findings demonstrating a correlation between the frequency of tumor cell engraftment and tumor grade in human breast tumors and melanomas [22], [54]. Hence differences in the sensitivity of the transplantation assays, the quality of the transplanted tumor cell preparations, the use of Matrigel to facilitate tumor cell engraftment, and/or perhaps the extent of tumor progression may account for the disparate TIC frequencies reported by us and others in the mammary tumors of the same or similar models.

The question arises as to whether the mammary tumors occurring in the transgenic mice we examined follow the cancer stem cell model especially in light of their high TIC fraction. All the mouse breast cancer models studied previously with the sole exception of the MMTV-Neu transgenic mice have been suggested to follow this model [17]–[20]. Consistent with the cancer stem cell hypothesis tumors arising in either the MMTV-mT or MMTV-Wnt-1/β-catenin transgenic strains are heterogeneous comprising cells that express markers of either the luminal or myoepithelial lineages, the two principal cell lineages in the mouse mammary gland. By contrast, the tumors of the MMTV-Neu model are seemingly homogeneous comprising predominantly luminal-lineage restricted cells [24], [25]. Accordingly it has been suggested that the mT and Wnt-1 induced tumors originate from stem cells or bipotent progenitor cells, whereas the Neu-induced tumors may emerge from luminal-restricted progenitor cells [31], [55].

In keeping with the cellular heterogeneity of the tumors in the mouse models, the use of antibodies to mammary epithelial stem cell surface markers together with FACS have permitted sorting mammary tumor cells into tumorigenic and non-tumorigenic fractions. A very minor tumorigenic cell population can be separated from the bulk, non-tumorigenic tumor cell fraction using FACS in the MMTV-Wnt-1 model, analogous to the MMTV-β-catenin model we studied [17], [19]. By contrast, Vaillant et al reported that whereas the tumors from the MMTV-Neu model, identical to that which we investigated, also comprised a relatively minor TIC fraction (∼1%), the TIC could not be similarly separated from the bulk tumor cells using antibodies to CD61, CD29 and CD24, leading these investigators to suggest that these tumors do not follow the cancer stem cell model [19]. Whether other combinations of cell surface markers might permit separation of TIC from the bulk tumor cell population in tumors of the MMTV-Neu transgenic strain has not been reported.

In light of the very high TIC frequency in the mouse mammary tumors we studied, the unavailability of markers that might facilitate fractionation of TIC from non-tumorigenic cells in the MMTV-Neu model [19] and the finding that FACS compromises TIC survival [43], we did not attempt to sort the bulk tumor cell populations of the models we studied into tumorigenic and non-tumorigenic fractions. Instead we asked whether other means might be used to determine whether the tumors we studied conformed to the cancer stem cell model. To this end we enquired whether conditions that favor normal stem cell self-renewal or differentiation might influence TIC frequency. A central tenet of the cancer stem cell model is that quasi-stable epigenetic differences distinguish TIC from their differentiating non-tumorigenic descendants [9], [47].

We found that the TIC frequency of the mammary tumors in the MMTV-Neu model could be maintained by culturing freshly isolated tumor cells in chemically-defined, serum-free medium, but that propagating these cells for a similar period and passage number in serum-containing medium reduced the TIC fraction by roughly 4 orders of magnitude. It is noteworthy that only 1% of primary tumor cells form tumorspheres, and that this fraction of sphere-forming cells does not vary during serial passage [56]. Hence the vast majority of the dispersed tumor cells do not survive plating in serum-free medium and because cell proliferation during sphere formation subsides substantially after 3–4 days in culture under the growth conditions we employed, there is no net expansion of the total tumor cell population during serial passage of bulk tumorsphere cultures. The growth factors in serum-free medium (FGF-2 and EGF) may sustain self-renewal of TIC and/or limit their differentiation into highly proliferative progenitor-like cells. By contrast, the plating efficiency of primary tumor cells varied between 10%–30% in serum-containing medium and the cells proliferated resulting in net expansion of the starting cell population by ∼10-fold during their initial serial passage. The latter findings are consistent with the possibility that serum factors initiate a differentiation program in TIC resulting in the transient proliferation of their progenitor like descendants, which culminates in their aberrant differentiation and consequent loss of proliferative and tumorigenic capacity. The latter hypothesis is consistent with the observation that normal mammary epithelial stem/progenitor cells can be propagated as mammospheres in serum-free medium, and undergo a differentiation program when propagated in serum-containing medium [36]–[38], [48]. However, we have no evidence that the differentiation of TICs leads to their loss of tumorigenicity *in vitro* and cannot rule out the possibility that TICs simply do not survive in serum-containing medium.

It seems unlikely that TIC were diluted by the expansion of non-tumorigenic cells in serum-containing medium because the net increase in total cell number was roughly 10-fold, whereas the decline in TIC frequency was 10,000 fold or greater. The possibility that fibroblasts present in the primary tumor cell preparations overgrew the epithelial tumor cells during their propagation in serum-containing medium seems unlikely because the vast majority of the cells growing in serum-containing medium appeared to be epithelial in morphology independent of their passage history. Because the tumor cells were cultured *in vitro* in either growth condition for only 12 days it also seems unlikely that genetic changes account for their wide difference in TIC frequency.

At this juncture is not clear what specific factors account for the difference in TIC fraction between tumors arising in the same mouse models reported by different laboratories, or whether the various models studied follow the cancer stem cell model. Whereas resolving these matters have therapeutic implications and consequently needs to be critically examined, the high TIC frequency in mouse mammary tumors and the capacity to maintain TIC-rich or TIC-depleted tumor cell populations *in vitro* may afford an opportunity to identify TIC biomarkers and TIC-targeted therapeutic agents, especially in view of the technical limitations inherent in sourcing human breast TIC to this end. Indeed we have used the mouse mammary tumorspheres and corresponding normal mouse mammospheres to identify TIC-selective compounds [56], [57].

## Conclusions

Our results demonstrate that mouse mammary tumors, including those arising in transgenic strains thought to conform to the cancer stem cell model, can comprise a very high TIC frequency that approach 50% in some tumors. Moreover, we show that a similarly high TIC frequency can be maintained by propagating the tumor cells in serum-free medium as non-adherent tumorspheres. By contrast, culturing the tumor cells for an identical period in serum-containing medium resulted in a substantial reduction in TIC frequency. These various tumor cell sources provide a rich source of tumorigenic or non-tumorigenic tumor cells for comparative analyses.

## Supporting Information

Figure S1 Tumors arising from transplant of tumor cells propagated in serum-containing medium recapitulate the histology the parental tumor. (**A**), Histology of a primary mammary tumor from an MMTV-Neu transgenic mouse. (**B**), Histology of a tumor seeded by transplant of tumor cells from the tumor shown in panel A that had been propagated *in vitro* in serum-containing medium. H&E staining of tumor sections illustrates the cytoarchitecture characteristic of Neu-induced tumors. Scale bar (inset) represents 40 µm in all panels.
(TIF)
