# Supplementary figures and images for: Single Unpurified Breast Tumor-Initiating Cells from Multiple Mouse Models Efficiently Elicit Tumors in Immune-Competent Hosts
Source: PLoS One. 2013 Mar 26;8(3):e58151. doi: 10.1371/journal.pone.0058151 (PMC3608640; doi:10.1371/journal.pone.0058151)

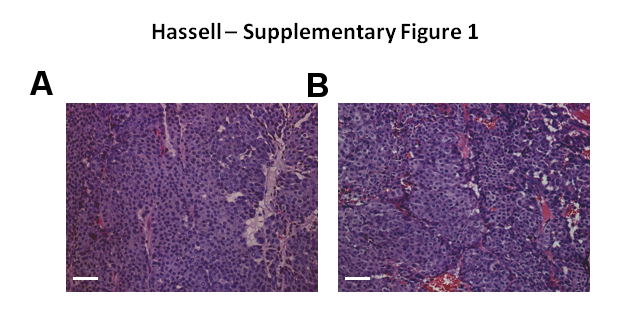

Supplement: Figure S1 — Tumors arising from transplant of tumor cells propagated in serum-containing medium recapitulate the histology the parental tumor. (A), Histology of a primary mammary tumor from an MMTV-Neu transgenic mouse. (B), Histology of a tumor seeded by transplant of tumor cells from the tumor shown in panel A that had been propagated in vitro in serum-containing medium. H&E staining of tumor sections illustrates the cytoarchitecture characteristic of Neu-induced tumors. Scale bar (inset) represents 40 µm in all panels. (TIF) [file pone.0058151.s001.tif]
